# Stroke survivor views on ambulance redirection as a strategy to increase access to thrombectomy in England

**DOI:** 10.29045/14784726.2024.6.9.1.1

**Published:** 2024-06-01

**Authors:** Abigail Alton, Darren Flynn, David Burgess, Gary A. Ford, Chris Price, Martin James, Peter McMeekin, Michael Allen, Lisa Shaw, Phil White

**Affiliations:** Newcastle University ORCID iD: https://orcid.org/0000-0002-9983-080X; Northumbria University ORCID iD: https://orcid.org/0000-0001-7390-632X; North East and North Cumbria Stroke Patient & Carer Panel, CRN North East and North Cumbria ORCID iD: https://orcid.org/0009-0003-3248-4601; University of Oxford ORCID iD: https://orcid.org/0000-0001-8719-4968; Newcastle University ORCID iD: https://orcid.org/0000-0003-3566-3157; Royal Devon & Exeter Hospital/University of Exeter Medical School; NIHR South West Peninsula Applied Research Collaboration ORCID iD: https://orcid.org/0000-0001-6065-6018; Northumbria University ORCID iD: https://orcid.org/0000-0003-0946-7224; University of Exeter Medical School & NIHR South West Peninsula Applied Research Collaboration ORCID iD: https://orcid.org/0000-0002-8746-9957; Newcastle University ORCID iD: https://orcid.org/0000-0002-9931-7774; Newcastle University ORCID iD: https://orcid.org/0000-0001-6007-6013

**Keywords:** pre-hospital care, qualitative research, stroke, thrombectomy

## Abstract

**Introduction:**

Intravenous thrombolysis and mechanical thrombectomy are effective time-sensitive treatments for selected cases of acute ischaemic stroke. While thrombolysis is widely available, thrombectomy can only be provided at facilities with the necessary equipment and interventionists. Suitable patients admitted to other hospitals require secondary transfer, causing delays to treatment. Pre-hospital ambulance redirection to thrombectomy facilities may improve access but treatment eligibility cannot be confirmed pre-hospital. Some redirected patients would travel further and be displaced without receiving thrombectomy. This study aimed to elicit stroke survivor and carer/relative views about the possible consequences of introducing a conceptual, idealised ambulance redirection pathway.

**Methods:**

Focus groups were undertaken using a topic guide describing four hypothetical ambulance redirection scenarios and their possible consequences: earlier treatment with thrombectomy; delayed diagnosis of non-stroke ‘mimic’ conditions; delayed thrombolysis treatment; and delayed diagnosis of haemorrhagic stroke. Meetings were audio recorded, transcribed verbatim and data analysed thematically using emergent coding.

**Results:**

Fifteen stroke survivors and carers/relatives participated in three focus groups. There was wide acceptance of possible low-risk consequences of ambulance redirection, including extended travel time, being further from home and experiencing longer hospital stays. Participants were more uncertain about higher-risk consequences, including delays in diagnosis/treatment for patients unsuitable for thrombectomy, but remained positive about ambulance redirection overall. Participants rationalised acceptance of higher-risk consequences by recognising that redirected patients would still access appropriate treatment, even if delayed. In addition, acceptance of ambulance redirection would be increased if there were robust clinical evidence showing net benefit over secondary transfer pathways.

**Conclusions:**

Participant views were generally supportive of ambulance redirection to facilitate access to thrombectomy. Further research is needed to demonstrate overall benefit in an NHS context.

## Introduction

Stroke is a vascular medical emergency due to cerebral ischaemia (85%) or haemorrhage (15%), which is experienced by >100,000 people in the UK annually (Patel et al., 2020). Over half of all survivors live with long-term disability (Stroke Association, 2022), but the outlook can be significantly improved by rapid delivery of targeted interventions (Intercollegiate Stroke Working Party, 2023; National Institute for Health and Care Excellence, 2022). To access these treatments, national guidelines recommend that patients with acute stroke symptoms should be identified by ambulance clinicians using a standard assessment, such as the Face Arm Speech Test (FAST), and taken to the nearest stroke unit without delay (Intercollegiate Stroke Working Party, 2023; Joint Royal Colleges Ambulance Liaison Committee, 2022).

Following hospital confirmation of ischaemic stroke, intravenous thrombolysis (IVT) within 4.5 hours of symptom onset for suitable patients reduces the risk of long-term disability (Wardlaw et al., 2014). Acute thrombotic blockage of a main artery (large vessel occlusion or LVO) is the mechanism of ischaemia for up to 30% of cases, which is associated with a high probability of severe disability despite IVT (Lakomkin et al., 2019). For selected LVO patients, the addition of mechanical thrombectomy provides greater efficacy than IVT alone (Balami et al., 2015), but the necessary specialist facilities and clinical expertise are only available at one in four stroke hospitals across England (Allen et al., 2019, 2022). As a result, three quarters of stroke patients eligible for treatment require secondary transfer by emergency ambulance from their local hospital to the closest thrombectomy facility, a process called ‘drip and ship’ because IVT is often given prior to transfer. Although this enables access to thrombectomy for the largest number of patients, it typically leads to a treatment delay of 1‒2 hours and poorer health outcomes when compared to patients directly admitted to a thrombectomy facility as their nearest stroke hospital (Allen et al., 2019, 2022).

Ambulance redirection is one proposed strategy for improving access to thrombectomy and LVO stroke outcomes (Allen et al., 2022; Mazya et al., 2020; Pérez de la Ossa et al., 2022). In an ambulance redirection pathway, patients identified with possible LVO stroke during initial ambulance assessment would be taken directly to the nearest thrombectomy facility, bypassing closer local stroke units. Although various symptom assessments have been proposed for pre-hospital identification of possible LVO stroke patients to trigger an ambulance redirection pathway, these are not currently recommended in national guidelines, mainly because of concerns about their accuracy. A systematic review involving 36 studies (including only four from the pre-hospital setting) demonstrated that with a positive prediction assessment, the probability of LVO might be only 50%‒60%, while the probability of LVO with a negative test could still be ≥10% (Smith et al., 2018). This would mean many non-LVO patients being diverted to thrombectomy facilities, with some true LVO patients not being recognised. Other potential issues with an ambulance redirection pathway include (Price et al., 2022): (i) due to the imperfect accuracy of pre-hospital clinical assessment in recognising stroke and differentiating between stroke subtypes, ambulance redirection may lead to delayed diagnosis and treatment of non-LVO conditions (e.g. haemorrhagic stroke, non-stroke mimic conditions); (ii) ambulance redirection may result in delayed IVT for some stroke patients found to be unsuitable for treatment with thrombectomy on arrival at a centre; (iii) ambulance redirection is likely to displace acutely unwell patients to hospitals further away from home, which may introduce challenges for patients and their relatives; (iv) ambulance redirection could increase the length of stay in hospital, due to delays associated with transferring redirected patients back to their local hospital.

When there are trade-offs relating to potential new healthcare delivery processes, understanding patient and public preferences helps to optimise the acceptability of decision-making by commissioners and service providers (Dirksen et al., 2013). This study therefore aimed to elicit stroke survivors’ and their carers’/relatives’ views about the possible consequences of an idealised ambulance redirection pathway intended to improve access to thrombectomy treatment.

## Methods

This qualitative study used a focus group approach to elicit views about ambulance redirection from stroke survivors and their carers/relatives, and is described in accordance with the Consolidated Criteria for Reporting Qualitative Research (COREQ) checklist (Tong et al., 2007). As an initial exploration of this topic, we did not utilise a specific theoretical framework to avoid the potential risk of theoretical interpretation impacting on the voice of the participants (Willig, 2017). Ethical approval was granted by Newcastle University Research Ethics Committee (12730/2020). The research team included a public representative, who assisted with the development of information materials, with explaining the purpose of the study to individuals who responded to invitations to participate and with interpretation of the main results.

### Recruitment and participants

A convenience sample of stroke survivors and carers/relatives was recruited from Integrated Stroke Delivery Networks (ISDNs) and regional stroke support groups in England. A named contact for each group was asked to distribute participant information sheets to their membership. Participant information sheets were designed to be accessible to stroke survivors with communication and comprehension issues (Parr et al., 2004). People who expressed an interest in participation were invited to attend an optional introductory meeting with a researcher (AA) and a patient, carer and public involvement (PCPI) representative (DB) to learn more about the research and ask any questions they had about participation. Informed written consent was obtained from participants prior to focus groups.

### Data collection

Focus groups were organised at a time agreeable to investigators and participants. Where possible, focus groups took place face to face at appropriate community venues, and were otherwise conducted remotely using Microsoft Teams video call software. Groups were facilitated by two experienced researchers (AA, a Research Assistant, and DF, a Professor of Applied Health and Social Care Research), with a PCPI representative (DB) in attendance.

**Figure 1:**
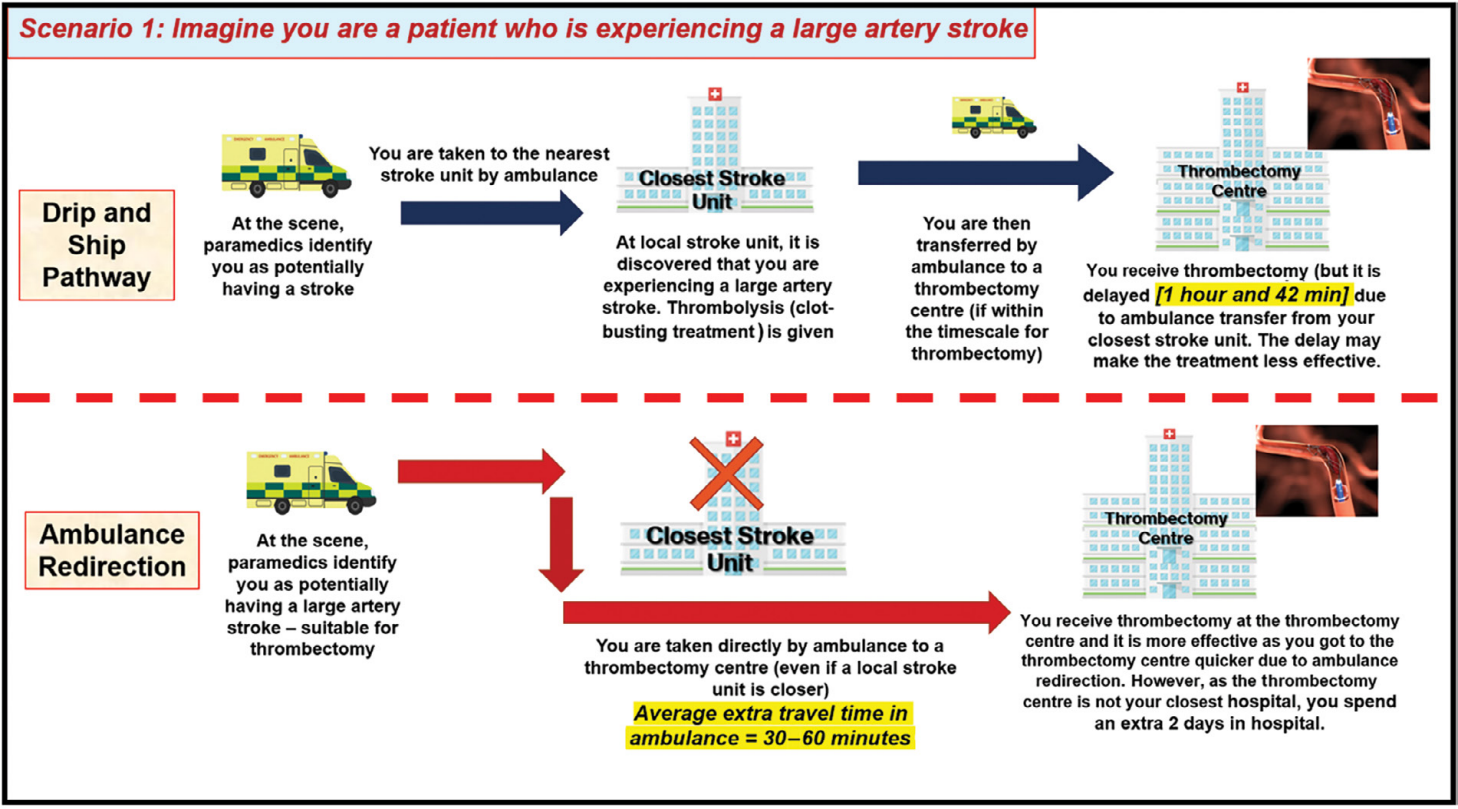
Figure 1. Focus-group scenario depicting trade-offs and potential outcomes for LVO patients under the drip and ship and ambulance redirection pathways.

**Figure 2:**
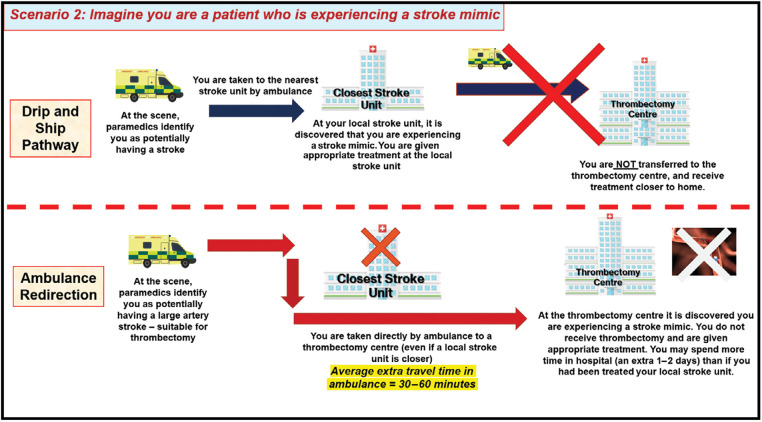
Figure 2. Focus-group scenario depicting trade-offs and potential outcomes for stroke-mimic patients under the drip and ship and ambulance redirection pathways.

**Figure 3:**
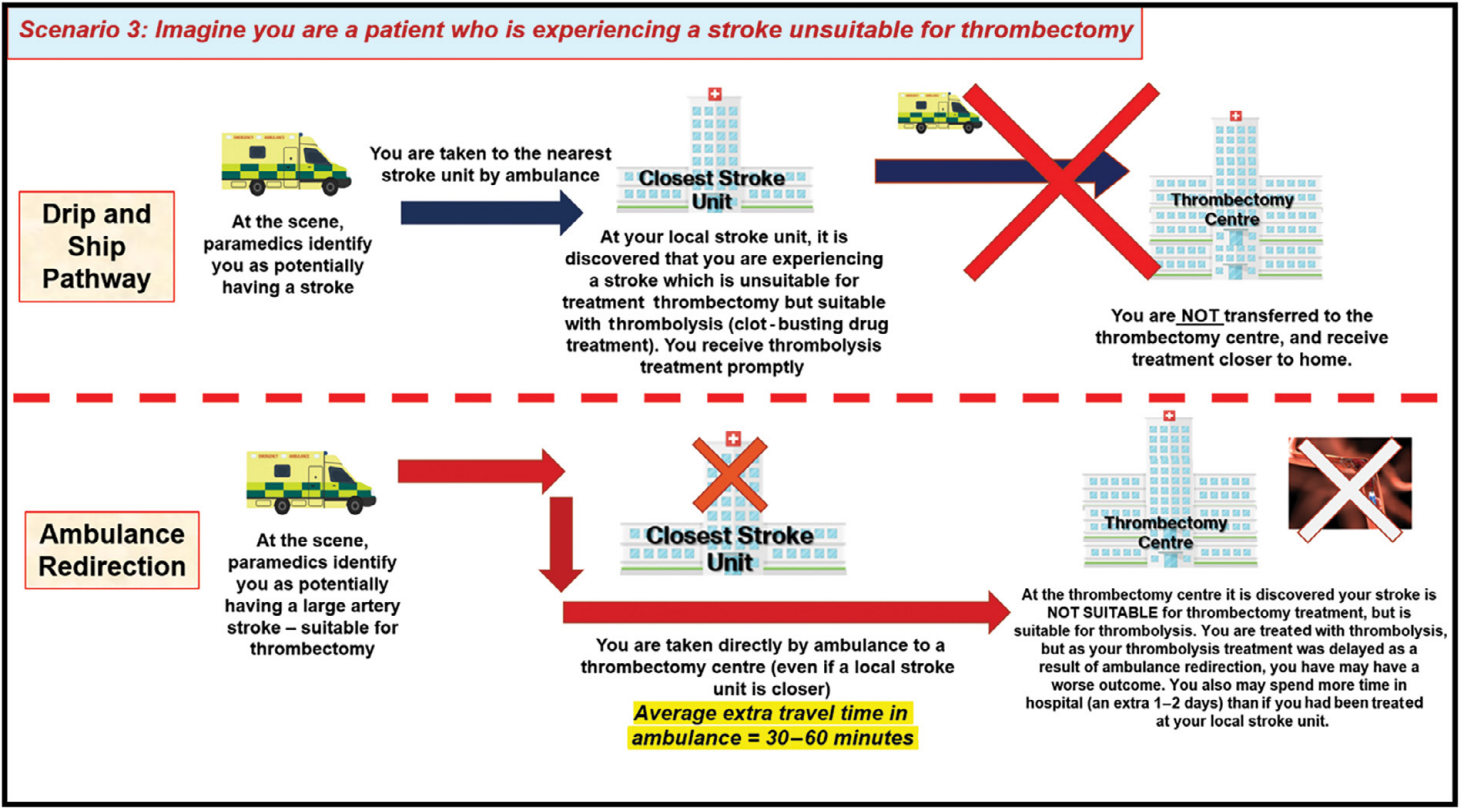
Figure 3. Focus-group scenario depicting trade-offs and potential outcomes for patients experiencing a stroke unsuitable for thrombectomy under the drip and ship and ambulance redirection pathways.

**Figure 4:**
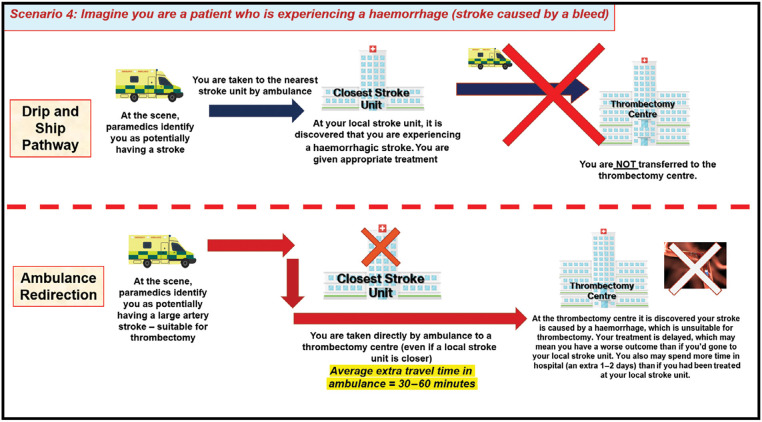
Figure 4. Focus-group scenario depicting trade-offs and potential outcomes for patients experiencing a haemorrhage under the drip and ship and ambulance pathways.

Focus group meetings consisted of a PowerPoint presentation to provide background information on ambulance redirection, followed by a plenary discussion with reference to a topic guide detailing four hypothetical scenarios developed to convey information and elicit views on the trade-offs associated with redirection versus secondary drip and ship transfer. These were: a patient receiving earlier thrombectomy due to redirection (Figure 1); delayed diagnosis of stroke mimic due to redirection (Figure 2); a patient unsuitable for thrombectomy receiving delayed thrombolysis due to redirection (Figure 3); and delayed diagnosis of haemorrhagic stroke due to redirection (Figure 4).

The focus group presentation also introduced the idea that several trade-offs could be influenced by how accurately LVO is identified in the pre-hospital setting, to initiate a discussion about acceptable levels of ‘correct’ and ‘incorrect’ ambulance redirection.

The number of focus groups conducted was based on practical considerations, as the project had limited funding and it was not possible to confirm data saturation, but it was agreed among the research team that key issues had already been identified before data collection ceased.

### Analysis

Focus-group meetings were audio recorded, transcribed verbatim and anonymised. The discussions generated following the presentation of each scenario across the focus groups were subjected to thematic analysis using emergent coding (Braun & Clarke, 2021). AA first coded the transcript data line by line to develop provisional codes across focus groups. DF independently coded the transcript data to increase the credibility of the analysis. Provisional themes arising from focus-group discussions on each scenario were discussed in meetings between AA and DF, and with other study authors who served as a forum to establish the credibility of the analysis.

## Results

Fifteen stroke survivors and carers/relatives were recruited. Three focus groups took place between June 2021 and October 2022. Two of these were held face to face with participants in Sunderland (N = 4) and Merseyside (N = 7). The third group was held virtually using Microsoft Teams with participants from South Yorkshire (N = 4). Focus groups lasted between 59 and 150 minutes.

Three themes were generated to represent similar views arising across scenario discussions:

1. Acceptability of lower-risk or short-term trade-offs associated with ambulance redirection; extended travel time, further distance from home and longer hospital stays.
2. Acceptability of potentially higher-risk or longer-term trade-offs associated with ambulance redirection; impact on delayed diagnosis and treatment of patients without LVO stroke.
3. Acceptability of ambulance redirection according to how accurately initial emergency assessment detects LVO.

### Theme 1: acceptability of lower-risk or short-term trade-offs associated with ambulance redirection: extended travel time, further distance from home and longer hospital stays

Participants consistently expressed across scenario discussions that lower-risk short-term trade-offs such as extended travel time, distance from home and extended hospital stays due to ambulance redirection would not be a concern for them or their family members in the context of an increased opportunity to receive thrombectomy sooner. Participants expressed that their main priority would always be health outcomes:

*The most important part is the outcome, it’s what you’re left with.* (Male stroke survivor)

*I think we’d do anything to get better. You say about being further away from your family – I’ve never felt so far away from my family as I do now, because of the way I am because of my stroke. My grandkids – I can’t play with them – I can’t play football with them, I can’t play superman with them.* (Male stroke survivor)

These views were held even when the scenario being discussed involved a patient experiencing greater travel time, distance from home and length of hospital stay when it turned out that thrombectomy was not appropriate. One participant highlighted that extra travel time in an ambulance would be unlikely to impact on acute stroke patients’ experiences of care, as many recalled not being cognisant at the time of their stroke.

*The thing about extra time in the ambulance – I don’t think you would realise anyway if you were going through a stroke or a stroke mimic that you were spending a long time in the ambulance – I don’t think that’s on your agenda really.* (Male stroke survivor)

### Theme 2: acceptability of potentially higher-risk or longer-term trade-offs associated with ambulance redirection: impact on delayed diagnosis and treatment of patients without LVO stroke

Participants were initially unsure on their views about the potential of receiving delayed diagnosis and treatment when their symptoms were due to non-stroke-mimic conditions and stroke without LVO. This reflected their focus on long-term health outcomes, especially as delayed IVT could have a negative impact.

*But at that point, do you then say ‘Give them thrombolysis and then ship them’ or do you say ‘Ship them and then give them thrombolysis’?* (Male stroke survivor)

Despite this, even when considering the potential impact of delayed IVT treatment for ischaemic stroke, participants remained in favour of ambulance redirection and rationalised this viewpoint in several different ways. Participants recognised the value of thrombectomy and expressed an altruistic view favouring ambulance redirection because it could result in the most severely affected patients with LVO having a better chance of receiving effective treatment. Participants also recognised that delayed IVT would not preclude access to thrombolysis completely, as this would still be available at thrombectomy hospitals. Finally, participants expressed that if they did have a worse outcome due to delayed IVT because of ambulance redirection, they would not be cognisant of what a ‘better outcome’ might have been:

*Let’s just say that I didn’t have the thrombolysis, and I were took straight for thrombectomy, and I didn’t need that, but they did the thrombolysis then, and let’s just say I’ve got vision loss in one eye, I’ve got a weak arm and a weak leg. Let’s just say that was slightly worse than it is now – I wouldn’t know any different. So, I’d think I were better than I was when I went in. Because I couldn’t speak, I couldn’t move my left side, I thought the thrombolysis were a miracle drug that helped me along. Now if I hadn’t have had that straight away, let’s just say my left arm were even weaker, I would never know that it could’ve been better. Does that make sense to everyone?* (Female stroke survivor)

Consistent with their views about delayed IVT, participants were prepared to accept the risk that ambulance redirection might result in delayed treatment for haemorrhagic stroke and non-stroke mimics, as long as they would still receive appropriate care following arrival at a thrombectomy hospital. Additionally, when considering possible delayed diagnosis and treatment of patients due to ambulance redirection, many participants expressed a preference to trust these high-risk decisions to those with specialist knowledge and expertise:

*You do what you think is best. I don’t know anything about it. It’s your decision. You know. A plumber can’t be telling a doctor what to do.* (Male stroke survivor)

Several participants also expressed that they would be more reassured about accepting the higher-risk trade-offs of ambulance redirection if clinical evidence demonstrated that it was better for the overall population compared to the standard secondary transfer emergency stroke care pathway:

*Surely someone in the know has got to work out what is best for the majority and go with that.* (Female stroke survivor)

### Theme 3: acceptability of ambulance redirection according to how accurately initial emergency assessment detects LVO

As the ability to identify LVO influences the value of ambulance redirection, focus groups involved a discussion about what level of accuracy for diagnosis of LVO would be acceptable. Understandably, all participants expressed that the highest possible accuracy would be preferable to prevent erroneous redirection. However, as participants felt that the relative risks associated with erroneous redirection in the ambulance redirection paradigm were a worthwhile trade-off for the chance of receiving thrombectomy if needed (and that those wrongly redirected would still receive appropriate care and treatment, albeit delayed), some participants were willing to consider accuracy levels as low as 40%, although this differed substantially, ranging from 40% to 90%:

*If it’s got to be better than chance it’s got to be 70%, shouldn’t it, really.* (Male stroke survivor)

*More than 50/50? Maybe 60/40 or something? But if you’re wrongly identified, is it really going to cause you that much of a problem, going down that route?* (Male stroke survivor)

## Discussion

This is the first study to explore the views of stroke survivors and carers/relatives regarding possible trade-offs between ambulance redirection and usual care (drip and ship) pathways to access to thrombectomy for LVO stroke in an NHS setting. Our data indicated that participants are willing to accept consequences such as extra travel time in an ambulance, being cared for at a greater distance from home and staying longer in hospital, in exchange for a better long-term outcome from thrombectomy. This view was underpinned by an expectation that people with non-LVO stroke diagnoses would still receive appropriate (albeit potentially delayed) treatment and care.

These findings corroborate previous work suggesting that patients prioritise the quality of care and health outcomes over inconvenience incurred by being treated at a hospital further away from home (Dirksen et al., 2013). For stroke, the results are consistent with a previous online survey of 147 public participants (27 stroke survivors, 51 relatives/carers of stroke survivors and 69 other members of the public), where 97% of respondents accepted hospital transfer to access thrombectomy and 75% were prepared to travel up to 30 miles for treatment (Price et al., 2022). Although extended distance from home may create logistical and psychological challenges for patients and their relatives, services could take steps to ameliorate these issues, for example providing flexibility around visiting times for relatives travelling considerable distances or grants for travel costs (Moynihan et al., 2013) or through development of rapid repatriation pathways so that displacement is only for a short time (Griffin et al., 2020).

There was more uncertainty around the acceptability of trade-offs posing a higher risk of negative impacts on outcomes (delayed treatment with thrombolysis for ischaemic stroke and delayed diagnosis and treatment of haemorrhagic stroke and stroke mimics). When considering these scenarios, participants were still supportive of ambulance redirection, but often expressed a view that pathway decisions may best be made by those with specialist knowledge and expertise. This finding is consistent with previous work in the context of hyper-acute stroke, whereby patients and their families often prefer to leave emergency high-risk decisions in the hands of expert clinicians and service planners (Busetto et al., 2020; Slot & Berge, 2009). However, it has also been reported that preferences vary among patients and carers regarding their information needs and involvement in clinical decision-making (Davis et al., 1999; Flynn et al., 2015; Schoenfeld et al., 2018). It is important that high-quality research is undertaken about the overall impact of ambulance redirection on health outcomes, so that directly relevant evidence is available about higher-risk consequences for those patients and carers who wish to form their own view.

Real-world clinical evidence supporting a policy shift towards ambulance redirection pathways is currently lacking. Previous modelling work has used assumptions about LVO symptom recognition, travel times and delivery of emergency stroke treatments in an NHS context to show that a theoretical ambulance redirection pathway could lead to an average improvement in time to thrombectomy of 80 minutes, but predicted an average delay in IVT of 8 minutes and a large increase in admissions to thrombectomy centres (Allen et al., 2019). The Spanish ‘RACECAT’ randomised controlled trial examined the impact of ambulance redirection for patients with paramedic-identified LVO symptoms, and found that there was no net benefit for the health of the stroke population overall even though ambulance redirection patients with LVO received thrombectomy 56 minutes faster than those transferred from local stroke units (Pérez de la Ossa et al., 2022). The Stockholm Stroke Triage Study (Mazya et al., 2020) also observed a significantly reduced time from symptom onset to thrombectomy by 69 minutes when ambulance patients were selected for ambulance redirection by stroke clinicians at one thrombectomy centre during a pre-hospital telephone assessment. However, as this was a quality-improvement project in a single city, which did not randomise patients to ambulance redirection or usual care, the results should not be automatically generalised to other settings.

Focus-group participants recognised that the likelihood of suspected stroke patients experiencing the different ambulance redirection pathway trade-offs will depend upon the accuracy of pre-hospital LVO identification. For this initial exploration into public acceptability of the ambulance redirection concept, we did not specify how LVO stroke identification would be attempted, but participant views were mixed about the acceptability of different accuracy levels. In the RACECAT trial, a paramedic-completed symptom score had a positive predictive value of approximately 50% for finding LVO (Pérez de la Ossa et al., 2022), while real-world hospital clinician selection of ambulance patients in Stockholm resulted in correct LVO identification for 40% of ambulance redirection cases (Mazya et al., 2020). Collectively, the available evidence, combined with the views of the participants in this study, indicates a clear need for further development and robust evaluation of assessment tools, ambulance redirection care pathways and/or diagnostic tests with greater specificity to identify LVO stroke.

Finally, it is important to acknowledge that the views presented reflect a small group of volunteers, who may have chosen to participate because of a particular interest in the topic, and there should be caution in generalising the findings. Efforts were made to seek volunteers from around the UK but for practical reasons it was not possible to purposively select individuals from all regions, and future research should consider whether opinions vary according to demographic and/or geographical differences. This was an initial exploration of public views about ambulance redirection for LVO stroke, and the scenarios presented were idealised rather than reflecting the possible real-world impact. It was also not possible within the limitations of the project methodology and timeline to explore more nuanced views, including the relative tolerances that participants might have for risks according to increasingly complex scenarios, such as extended ambulance redirection travel times resulting in a complete loss of IVT opportunity.

## Conclusion

Stroke survivors and their carers/relatives provided generally positive views about ambulance redirection for facilitating access to thrombectomy, although there was some uncertainty about higher-risk consequences such as delayed IVT, and an acceptable level of accuracy for pre-hospital LVO identification. Policymakers, clinicians and the public now require robust clinical evidence demonstrating the benefit and consequences of ambulance redirection for the wider suspected stroke population in an NHS context.

## Acknowledgements

We would like to express our thanks to the following individuals and organisations: North East Stroke Service User Voices, South Yorkshire and Bassetlaw Integrated Stroke Delivery Network Patient Public and Carer Panel, Merseyside Life After Stroke Group.

## Author contributions

All authors contributed to the design of the study. AA, DF and DB facilitated the focus groups. AA and DF analysed data with input from DB. AA and DF wrote the first draft of the manuscript. All authors contributed to the drafting process and approved the submission of the final version of the manuscript. CP acts as the guarantor for this article.

## Conflict of interest

None declared.

## Ethics

Ethical approval was granted by Newcastle University Research Ethics Committee (reference number: 12730/2020). Consent was obtained for participation.

## Funding

This project was funded by the National Institute for Health and Care Research: Programme Development Grant NIHR201692. The views expressed are those of the author(s) and not necessarily those of the NIHR or the Department of Health and Social Care.
